# Temperature sensing by the calcium-sensing receptor

**DOI:** 10.3389/fphys.2023.1117352

**Published:** 2023-02-02

**Authors:** Sarah C. Brennan, Hee-chang Mun, Leigh Delbridge, Philip W. Kuchel, Arthur D. Conigrave

**Affiliations:** ^1^ School of Life and Environmental Sciences, Faculty of Science, The University of Sydney, Sydney, NSW, Australia; ^2^ Charles Perkins Centre, University of Sydney, Sydney, NSW, Australia; ^3^ Department of Surgery, Mater Hospital, North Sydney, NSW, Australia

**Keywords:** calcium sensing receptor, temperature sensing, intracellular calcium oscillations, protein kinase C, protein phosphatase, GPCR

## Abstract

Whether GPCRs support the sensing of temperature as well as other chemical and physical modalities is not well understood.

**Introduction:** Extracellular Ca^2+^ concentration (Ca^2+^_o_) modulates core body temperature and the firing rates of temperature-sensitive CNS neurons, and hypocalcemia provokes childhood seizures. However, it is not known whether these phenomena are mediated by Ca^2+^_o_-sensing GPCRs, including the calcium-sensing receptor (CaSR). In favor of the hypothesis, CaSRs are expressed in hypothalamic regions that support core temperature regulation, and autosomal dominant hypocalcemia, due to CaSR activating mutations, is associated with childhood seizures.

**Methods:** Herein, we tested whether CaSR-dependent signaling is temperature sensitive using an established model system, CaSR-expressing HEK-293 cells.

**Results:** We found that the frequency of Ca^2+^_o_-induced Ca^2+^_i_ oscillations but not the integrated response was linearly dependent on temperature in a pathophysiologically relevant range. Chimeric receptor analysis showed that the receptor’s C-terminus is required for temperature-dependent modulation and experiments with the PKC inhibitor GF109203X and CaSR mutants T888A and T888M, which eliminate a key phosphorylation site, demonstrated the importance of repetitive phosphorylation and dephosphorylation.

**Discussion and Conclusion:** CaSRs mediate temperature-sensing and the mechanism, dependent upon repetitive phosphorylation and dephosphorylation, suggests that GPCRs more generally contribute to temperature-sensing.

## Introduction

The calcium-sensing receptor (CaSR) is a class C G protein-coupled receptor (GPCR) that acts as a master regulator of calcium homeostasis with key actions in parathyroid, kidney and bone (Brown and MacLeod, 2001). It is also expressed widely in non-calcitropic tissues (i.e., tissues not directly involved in calcium metabolism) (Hannan et al., 2018) and, depending on the compartment (Conigrave et al., 2000a), acts as a multi-modal, multi-metabolic sensor that responds to endogenous ionic, nutrient and other chemical activators including Mg^2+^ (Brown et al., 1993), l-amino acids (Conigrave et al., 2000b; Conigrave et al., 2004; Liou et al., 2011; Wang et al., 2011) and glutathione and analogs (Wang et al., 2006; Broadhead et al., 2011), inhibitors including inorganic phosphate ions (Centeno et al., 2019), and key physicochemical modalities including ionic strength and pH (Quinn et al., 1998; Quinn et al., 2004; Campion et al., 2015). It is not known, however, whether the CaSR might also sense and respond to changes in temperature in the pathophysiological range as suggested by its expression in anterior preoptic neurons of the hypothalamus (Yano et al., 2004) and by the observation that activating mutations of the CaSR in type-I Autosomal Dominant Hypocalcemia (ADH) are associated with childhood febrile convulsions (Papadimitriou et al., 2001).

In response to small, physiologically relevant increases in Ca^2+^_o_ concentration, the CaSR mediates oscillations in cytoplasmic free Ca^2+^ (Ca^2+^_i_) in various cell types including transfected HEK-293 cells that translate CaSR mRNA (Breitwieser and Gama, 2001), parathyroid cells (Miki et al., 1995; Ridefelt et al., 1995), and colonic epithelial cells (Rey et al., 2010). In CaSR-expressing HEK-293 cells, elevated Ca^2+^_o_ induces low frequency (1—4 min^−1^) Ca^2+^_i_ oscillations (Young and Rozengurt, 2002) (review: (Breitwieser, 2006)) that are supported by the cyclical phosphorylation and dephosphorylation of the CaSR’s primary protein kinase-C (PKC) site at Thr-888 (T888), in its intracellular C-terminus (Bai et al., 1998; Jiang et al., 2002; McCormick et al., 2010). HEK-293 cells transfected with either of the CaSR mutants T888A or T888M, which eliminate the PKC dependent phosphorylation site, exhibit enhanced Ca^2+^_o_ sensitivity and support sustained increases in Ca^2+^_i_ rather than Ca^2+^_i_ oscillations (Davies et al., 2007; Lazarus et al., 2011).

Previously it was reported that the frequency of Ca^2+^_i_ oscillations was sensitive to a large change in temperature in CaSR-expressing HEK-293 cells (Breitwieser and Gama, 2001; Young et al., 2002); however, the possibility that the CaSR might act as a temperature-sensor has not been formally investigated. We considered whether the CaSR might exhibit temperature-sensitive properties that arise from the dual control of T888 phosphorylation *via* PKC and protein phosphatase activity, both of which are temperature sensitive (Sugiura et al., 2002; Kim et al., 2005; Chemin et al., 2007; Sohn et al., 2007). Thus, in the present study, we investigated whether the CaSR has temperature-sensing properties, as revealed by changes in the frequency and/or amplitude of Ca^2+^_o_-induced Ca^2+^_i_ oscillations in CaSR-expressing HEK-293 cells. We observed temperature-sensitive modulation of the frequency but not the amplitude of CaSR-mediated Ca^2+^_i_ oscillations together with roles for PKC and CaSR C-terminal residue T888 in supporting the mechanism.

## Materials and methods

### CaSR wild-type and mutant constructs and CaSR/mGluR1/mGluR1 chimeric receptor construct

The wild-type (WT) human CaSR cDNA was cloned between the Kpn I and Xba I sites of pcDNA3.1 (+). All mutants were generated in pcDNA3.1(+)-WT CaSR. The Quikchange II site-directed mutagenesis protocol was used to introduce the point mutations T888A and T888M, as described previously (Lazarus et al., 2011): briefly, pairs of complementary or overlapping primers (30–40 bases) were designed to encode the required mutation with flanking wild-type sequences of ∼15–20 bases. The template DNA was amplified for 18 cycles with Pfu Ultra II HS DNA polymerase (Agilent Technologies, USA). Following digestion of parent DNA with Dpn I, amplified DNA was transformed into DH5α *Escherichia coli* cells.

The hCaSR/rmGluR1/rmGluR1 chimeric receptor construct was described previously (Mun et al., 2004). It encodes a class C GPCR protein that contains the hCaSR VFT domain (residues 1–540) appended to the rat mGluR1alpha Cysteine-rich domain, heptahelical domain, and intracellular C-terminus (residues 524–1199).

The identities of all mutants were confirmed by DNA sequencing (Australian Genome Research Facility, Sydney, NSW, Australia).

### Cell culture and transfection

HEK-293 cells stably expressing the human CaSR (HEK-CaSR cells) or the hCaSR/rmGluR1/rmGluR1 (HEK-C/G/G) chimeric receptor were cultured in DMEM (Invitrogen, USA) supplemented with 10% fetal bovine serum (FBS) as described previously (Mun et al., 2004; Szekely et al., 2009). Cells were grown on 15-mm diameter glass coverslips in 24-well plates for microfluorimetry experiments.

For experiments with transiently expressing CaSR constructs, when the cells had reached 85%–95% confluency they were transfected with XtremeGENE HP transfection reagent (Roche, Germany): briefly, 0.5 μg samples of WT or mutant DNA and 3 μL of transfection reagent were diluted with 100 μL of DMEM and allowed to complex at RT for 15 min. The transfection solution was added to the cell cultures to a final concentration of 9.1% (v/v).

### Ca^2+^_i_ microfluorimetry

HEK-CaSR, HEK-C/G/G or transiently transfected HEK-293 cells were cultured on coverslips in 24-well plates and then loaded in the dark with Fura-2AM (5 μM) in physiological saline solution (PSS): 125 mM NaCl, 4 mM KCl, 0.1% (w/v) D-glucose, 1 mM MgCl_2_, 20 mM HEPES-NaOH (pH 7.45) that contained 1 mM CaCl_2_, 0.8 mM NaH_2_PO_4_, and 1 mg ml^−1^ BSA for 2 h at 37°C. The cells were stored in loading solution prior to experiments. Fura-2-loaded HEK-CaSR cells were then placed in a perifusion chamber in the light path of a Zeiss Axiovert 200M fluorescence microscope (×63 objective) and perifused with PSS containing various concentrations of Ca^2+^_o_. The PKC inhibitor GF109203X (Sigma-Aldrich) was used at a final concentration of 250 nM.

Fura-2-loaded HEK-CaSR cells were excited using a Lambda DG-4 150 Watt xenon light source (Sutter Instruments), with alternating wavelengths of 340 and 380 nm at 0.5s intervals, and the fluorescent cells were imaged at 510 nm. Each data set consisted of 10 regions of interest each containing an individual cell and digital images were captured using an AxioCam camera controlled by Stallion SB.4.1.0 PC software (Carl Zeiss).

The temperature in the perifusion chamber of the fluorescent microscope and therefore of fura-2-loaded cells was set by 1) a recirculating heat exchange system that delivered solutions of the desired temperature into the chamber *via* a peristaltic pump (Gilson Minipuls 2) and; 2) a Warner TC-344B Dual Channel Heat Controller that controlled the temperature of the microscope stage. To prevent heat losses, the inlet tubing to the microscope chamber was passed through a wide-bore 1.5 cm diameter tube containing water of the desired temperature that re-circulated *via* the external circuit of a thermostatically controlled water-bath. Using this system, the required perifusion solution temperatures were achieved by adjusting the demand temperatures of the water bath and the microscope stage as demonstrated by the temperatures measured in the effluent solutions (Supplementary Material). When the set temperature was adjusted, re-equilibration times of 1–2 min were observed.

### Human parathyroid cell preparation and perifusion

Human parathyroid cells were prepared by collagenase digestion of surgical specimens of normal parathyroid tissue as described previously (Conigrave et al., 2004; McCormick et al., 2010) according to ethics protocol 11_067 (Human Research Ethics Committee, St Vincent’s Health Network, Darlinghurst NSW 2010). Tissue samples were collected from the Mater Hospital (North Sydney) into MEM (Gibco #11575032; Ca^2+^ 1.2 mM) and transported to the laboratory on ice. After isolation and washing to remove collagenase by centrifugation (110 X *g*, 2 min), aliquots of cells (40,000—200,000) were loaded into columns to lie above a layer of Biogel P4 (Biorad #1504124) and below a layer of Sephadex G-25 medium (Sigma-Aldrich #G-25150) as described previously and perifused with physiological saline solution (NaCl 125 mM, KCl 4 mM, HEPES 20 mM, Na_2_HPO_4_ 0.8 mM, glucose 1.0 g L^−1^, MgCl_2_ 1.0 mM, pH 7.4) that contained bovine serum albumin 1 mg/ml (Sigma-Aldrich #A3059; protease-free) and various Ca^2+^ concentrations at 37°C as required. Perifusion columns were housed in metal holders that enabled full immersion and connections to be established upstream to the perifusion solutions and downstream to a peristaltic pump. The metal holders also protected the columns during transfer between waterbaths (see below). Samples were collected on ice and transferred to dry ice prior to storage at -80°C. Analysis for PTH was subsequently performed on a Siemens Immulite autoanalyzer (Royal Prince Alfred Hospital, NSW Health Pathology). To permit studies of the effect of temperature on Ca^2+^_o_-regulated PTH secretion, two water baths (one equilibrated at 35°C and one equilibrated at 38°C) were placed in close proximity and identical pre-equilibrated PSS-albumin solutions (i.e., containing the same Ca^2+^_o_ concentrations) were immersed in both baths to allow prompt temperature exchange (∼30 s) when required. To enable temperature changeovers, the perifusion pump was stopped briefly (20–30s) and perifusion columns were transferred to the neighboring bath at the new temperature (38°C or back to 35°C) where its upstream connections were established to the new temperature pre-equilibrated solution, and the pump restarted.

### Determination of integrated Ca^2+^_i_ responses and Ca^2+^_i_ oscillation frequency

Single-cell Ca^2+^_i_ mobilization data consisted of excitation ratios (F_340_/F_380_) plotted against experiment time (min). Where indicated, Ca^2+^_i_ responses from single cells were integrated with respect to time according to the ‘trapezoidal rule’, using the following equation:

$$\int_{x_{0}}^{x_{n}}f\left(x\right)dx\approx ∆x\;\left(\frac{y_{0}}{2}+y_{1}+\ldots y_{n-1}+\frac{y_{n}}{2}\right)$$ (1)

where Δ*x* is time interval between measurements (0.5 s) and *y* is F_340_/F_380_, to provide results in the form of integrated response units (IRUs) that were corrected for the baseline observed at control Ca^2+^_o_ (0.5 mM), as described previously (Brennan et al., 2015).

The fluorescence records from individual cells were processed to estimate the Ca^2+^_i_ oscillation frequency using wavelet analysis in a *Mathematica 9.0* script, as described previously (Szekely et al., 2009; Brennan et al., 2015). Frequency estimates from individual cells are expressed as means ± SEM.

### Statistical analyses

Statistical analyses were performed in GraphPad Prism version 8.0 using: Student’s unpaired *t*-test for direct comparisons between individual groups; one-way ANOVA with Dunnett’s post-test for comparisons between multiple groups, Linear Regression Analysis to evaluate the nature of the relationship between temperature and Ca^2+^_i_ oscillation frequency, and Analysis of Co-Variance (ANCOVA) for comparisons between regression lines.

## Results

### Temperature and frequency of CaSR-mediated Ca^2+^_i_ oscillations

**FIGURE 1:**
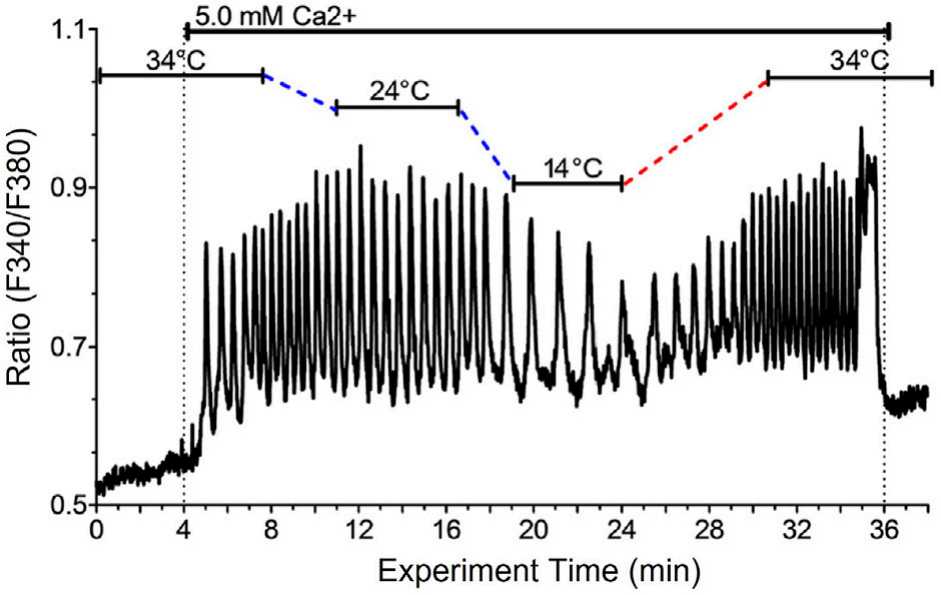
Reduced temperature reversibly lowers Ca2+i oscillation frequency in single HEK-CaSR cells exposed to elevated Ca2+o. HEK-CaSR cells were loaded with fura-2 AM then transferred to a microfluorescence apparatus and perifused with physiological saline solution (PSS) as described in the Methods section. After equilibration at control Ca^2+^_o_ (0.5 mM) the cells were exposed to a near-maximal Ca^2+^_o_ concentration (5.0 mM) at 34 °C and the temperature was then lowered progressively to 14 °C then restored to 34°C. Ca^2+^_o_ was then returned to the control level (0.5 mM).

In pilot experiments fura-2 loaded HEK-CaSR cells were perifused with PSS in the presence of a control sub-physiological temperature (34 °C) at low-baseline Ca^2+^_o_ (0.5 mM) and then exposed to an abrupt step-wise increase in Ca^2+^_o_ to a near-maximally effective Ca^2+^_o_ concentration, 5.0 mM. Once stable Ca^2+^_i_ oscillations had been established, the temperature was lowered to 14°C for 10–15 min and subsequently restored to the baseline temperature (34°C). At baseline temperature (34°C) and at 5.0 mM Ca^2+^_o_, the Ca^2+^_o_-induced Ca^2+^_i_ frequency was around 3 min^−1^ (Figure 1). As the temperature was lowered, however, we observed a substantial reduction in Ca^2+^_o_-induced Ca^2+^_i_ frequency to around 1 min^−1^ that promptly recovered to the control level as the temperature was restored to 34°C (Figure 1). Thus, an acute change in temperature induced a reversible change in Ca^2+^_o_-induced Ca^2+^_i_ frequency.

**FIGURE 2:**
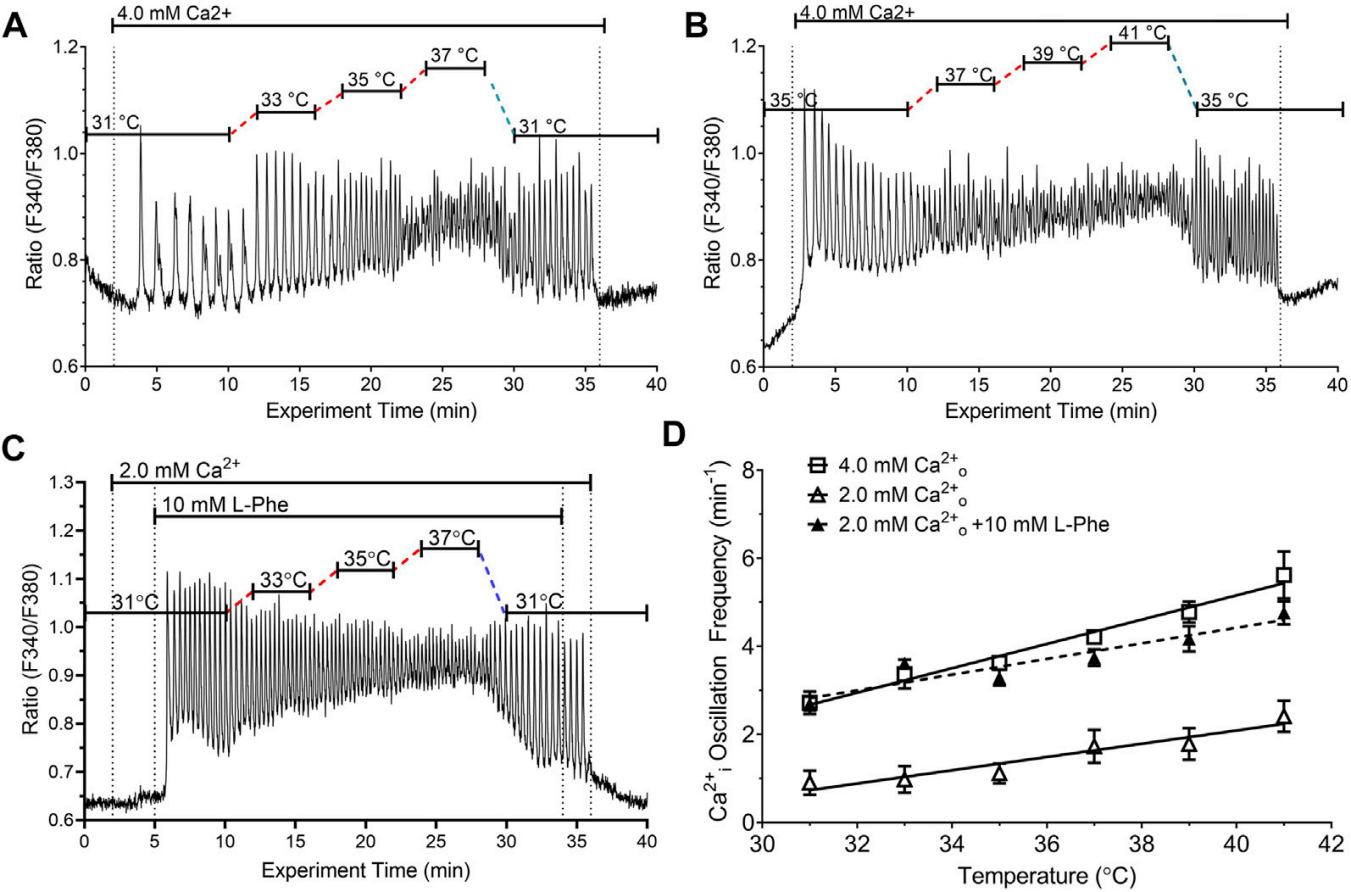
Impact of temperature on the frequency of Ca^2+^_o_-induced Ca^2+^_i_ oscillations in the absence or presence of CaSR positive modulator L-Phe. HEK-CaSR cells were loaded with fura-2 AM then transferred to a microfluorescence apparatus and perifused with physiological saline solution (PSS). After a brief period of equilibration at control Ca^2+^_o_ (0.5 mM), Ca^2+^_o_ was increased acutely to either (i) 4.0 mM or (ii) 2.0 mM in the absence or presence of 10 mM L-Phe. The temperature was adjusted as required using a customised heating system (see Methods). In **(A, B)**, Ca^2+^_o_ was increased acutely from 0.5 mM to 4.0 mM and the temperature was adjusted in the ranges 31°C–37°C and 35°C–41°C respectively. The dotted lines indicate 2 min periods of temperature adjustment. In **(C)**, the cells were exposed initially to 31°C at control Ca^2+^_o_ (0.5 mM) and the Ca^2+^_o_ level was then increased to 2.0 mM followed by the addition of L-Phe (10 mM). Panel **(D)** presents the combined results of 3-4 experiments in each case. Temperature-dependent changes in Ca^2+^_i_ frequency exhibited linear relationships for 4.0 mM Ca^2+^_o_ (open squares) and 2.0 mM Ca^2+^_o_ in the absence (open triangles) or presence (filled triangles) of 10 mM L-Phe.

We next determined the effects of stepwise changes in temperature in the pathophysiological range 31°C–41°C on fura-2 loaded HEK-CaSR cells that were exposed to increases in Ca^2+^_o_ from 0.5 mM to 4.0 mM. These Ca^2+^_o_ levels were selected based on the known Ca^2+^_o_ concentration-response behaviour of HEK-CaSR cells (Conigrave et al., 2000b; Brennan et al., 2015). The experiments were performed in two series with overlapping temperature ranges: 1) 31°C—37°C (Figure 2A); and 2) 35°C—41°C (Figure 2B). For the determination of Ca^2+^_i_ oscillation frequency at distinct temperatures, ratiometric traces obtained in individual cells were processed using wavelet analysis (73—157 cells from 8—17 individual experiments) and the data were pooled.

At the control Ca^2+^_o_ concentration (0.5 mM) all cells exhibited stable baseline Ca^2+^_i_ levels. When Ca^2+^_o_ was increased to 4.0 mM, however, around 95% of HEK-CaSR cells exhibited prompt oscillations in Ca^2+^_i_ across the temperature range 31°C–41°C. At normal physiological temperature (37°C) the Ca^2+^_i_ oscillation frequency was 4.21 ± 0.13 min^−1^ consistent with previous reports (Breitwieser and Gama, 2001; Brennan et al., 2015). In addition as temperature increased, Ca^2+^_i_ oscillation frequency increased progressively from 2.72 ± 0.26 min^−1^ at 31°C to 5.62 ± 0.54 min^−1^ at 41°C. The Q_10_ temperature co-efficient was around 2.1 and a linear relationship was observed between temperature and Ca^2+^_i_ oscillation frequency (Figure 2D) with a slope of 0.29 ± 0.03 min^−1^°C^−1^ (Table 1).

**TABLE 1:** Linear relationships between temperature and Ca^2+^_i_ frequency in HEK-CaSR cells exposed to Ca^2+^_o_ in the absence or presence of L-Phe Temperature: Ca^2+^_i_ frequency data were obtained in HEK-CaSR cells exposed to 1) near-maximal Ca^2+^_o_ (4.0 mM) or 2) submaximal Ca^2+^_o_ (2.0 mM) concentrations in the absence or presence of the amino acid L-Phe (positive modulator; 10 mM). The data were subjected to Linear Regression Analysis (GraphPad Prism 8). The slopes of the temperature: Ca^2+^_i_ frequency relationships are presented ±SE, together with the associated *r* ^*2*^ values. *p*_*1*_ values represent the probabilities that the calculated slopes were the same as zero. *p*_*2*_ values represent the probabilities that the calculated slopes for 2 mM Ca^2+^_o_ in the absence or presence of 10 mM L-Phe were the same as those observed in cells exposed to 4.0 mM Ca^2+^_o_. In all cases, the *p*-values demonstrate that the null hypotheses (no difference) were false.

Activators	Slope (min^−1^ °C^−1^)	r^2^	p_1_	p_2_
4.0 mM Ca2+	0.29 ± 0.03	0.52	<0.0001	-
2.0 mM Ca2+	0.16 ± 0.04	0.37	<0.005	0.0002
2.0 mM Ca2+ + 10 mM L-Phe	0.18 ± 0.03	0.43	<0.0001	0.0002

We further investigated the effect of temperature on Ca^2+^_i_ frequency at 2.0 mM Ca^2+^_o_, which lies below the concentration at which the CaSR is half-maximally activated (Conigrave et al., 2000b; Brennan et al., 2015), in the absence or presence of an endogenous L-amino acid modulator, L-Phenylalanine (L-Phe). As in the case of 4.0 mM Ca^2+^_o_, we observed linear relationships between temperature and Ca^2+^_i_ oscillation frequency at 2.0 mM Ca^2+^_o_ in both the absence and presence of L-Phe (10 mM), and L-Phe increased Ca^2+^_i_ oscillation frequency at all temperatures tested, without changing the slope of the temperature—Ca^2+^_i_ frequency relationship (Figures 2C, D; Table 1). Thus, Ca^2+^_i_ oscillation frequencies at 2.0 mM Ca^2+^_o_ exhibited a step-wise increase upon the addition of L-Phe but the calculated slope of the frequency-temperature relationship was unchanged (Figure 2D; Table 1). The Q_10_ temperature co-efficients were around 2.7.

### Effect of temperature on Ca^2+^_o_-induced integrated Ca^2+^_i_ responses

**FIGURE 3:**
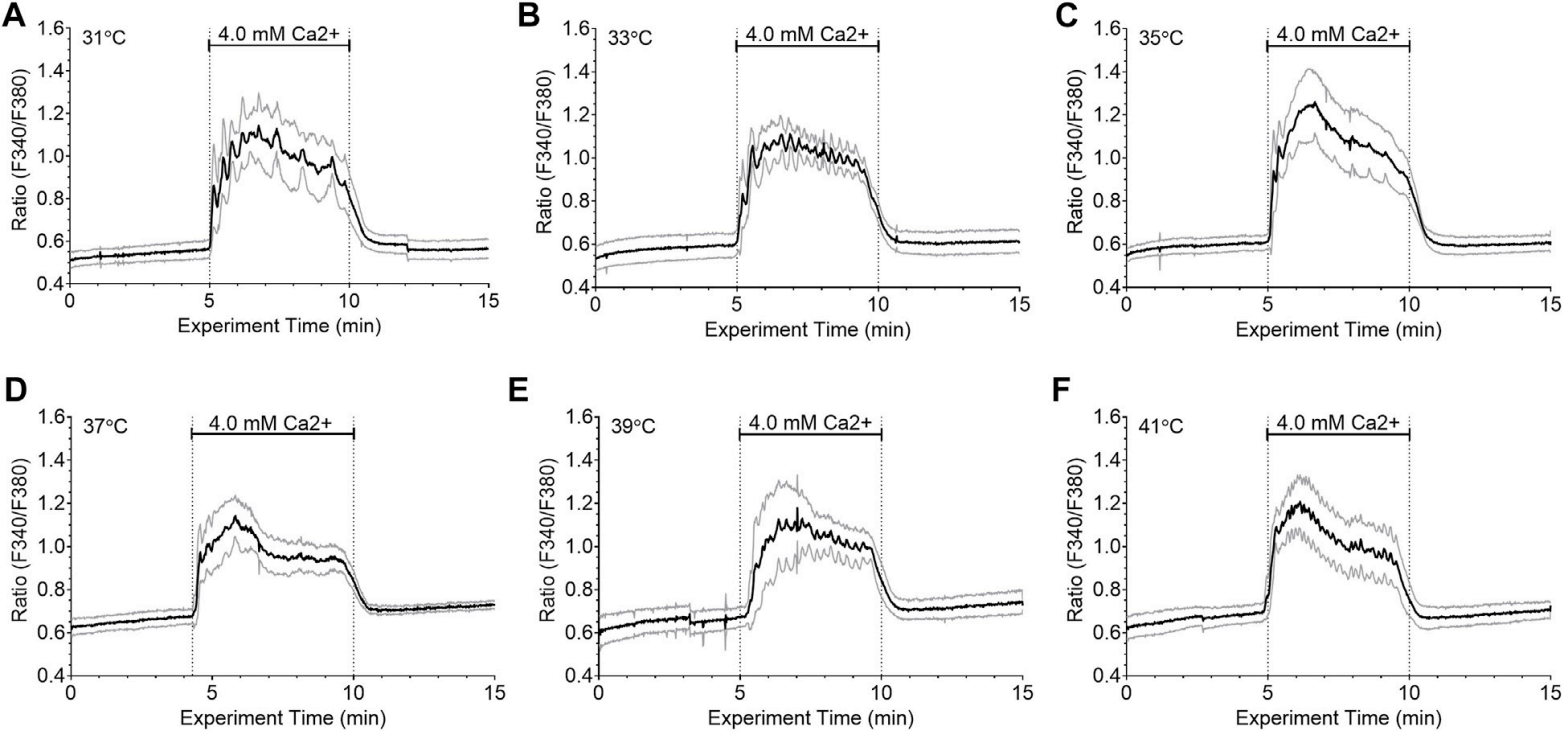
Lack of effect of temperature on integrated Ca^2+^_i_ responses in HEK-CaSR cells. HEK-CaSR cells were loaded with fura-2 AM at baseline Ca^2+^_o_ (0.5 mM) and adjusted to various set temperatures in the range 31°C–41°C. They were then exposed to elevated Ca^2+^_o_ (4.0 mM) for 5 min prior to return to baseline. Panels **(A—F)** show time-courses for the mean responses (solid black lines) as well as responses for means plus one SD and means minus one SD (grey lines) for 10 individual cells in each case. Temperature had no effect on the integrated Ca^2+^_i_ responses under the conditions of these experiments.

We next investigated whether temperature affected the integrated Ca^2+^_i_ responses to fixed Ca^2+^_o_ concentrations. Thus, HEK-CaSR cells were exposed to 4.0 mM Ca^2+^_o_ at different temperatures in the range 31°C–41°C (Figure 3). The Ca^2+^_i_ responses were integrated using data from 10 single cells per experiment. No significant differences were observed between the different temperatures. Wavelet analysis, however, revealed that the Ca^2+^_i_ frequency in each single cell was temperature dependent as described above, increasing from 2.99 ± 0.48 min^−1^ at 31°C, to 4.84 ± 0.70 min^−1^ at 37 °C, and to 6.56 ± 0.07 min^−1^ at 41°C. The results indicate that temperature modulated Ca^2+^_o_-induced Ca^2+^_i_ oscillation frequency but had no effect on the amplitudes of the Ca^2+^_i_ responses.

### Role of the CaSR C-terminus and residue T888 in temperature-dependent modulation

**FIGURE 4:**
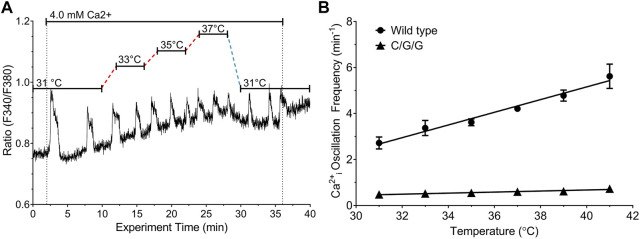
Effect of temperature on Ca^2+^_o_-induced Ca^2+^_i_ frequency in HEK-293 cells transfected with CaSR/mGluR1/mGluR1 chimeras. HEK-293 cells that stably expressed CaSR/mGluR1/mGluR1 chimeric receptors (HEK-C/G/G cells) or control HEK-CaSR cells were loaded with fura-2 AM and then exposed to 4.0 mM Ca^2+^_o_ as described in Materials and Methods. The temperature was adjusted as required in the range 31°C–37 °C. **(A)** Representative single cell Ca^2+^_i_ response to 4.0 mM Ca^2+^_o_ in the range 31°C–37°C in HEK-C/G/G cells. **(B)** Linear relationships between temperature and Ca^2+^_i_ frequency for HEK-CaSR (circles) and HEK-C/G/G cells (triangles) at 4.0 mM Ca^2+^_o_; several error bars are contained within the data points.

Cyclical phosphorylation and dephosphorylation of residue T888 in the CaSR’s intracellular C-terminus is required for Ca^2+^_o_-induced Ca^2+^_i_ oscillations (Davies et al., 2007; McCormick et al., 2010). In assessing the role of the CaSR C-terminus in temperature-dependent modulation of Ca^2+^_o_-induced Ca^2+^_i_ oscillations, we first tested the behavior of the chimeric receptor CaSR/mGluR1/mGluR1, which retains Ca^2+^_o_ sensing properties (Mun et al., 2004) but in which the CaSR’s Cysteine-rich domain, heptahelical domain and C-terminus are replaced by the corresponding domains of its class C receptor homolog, mGluR1α, which does not support Ca^2+^_i_ oscillations (Kawabata et al., 1998). In HEK-293 cells stably transfected with CaSR/mGluR1/mGluR1, we observed low frequency Ca^2+^_i_ transients as Ca^2+^_o_ increased from 0.5 mM to 4.0 mM but these responses did not exhibit temperature-dependent modulation (Figure 4).

**FIGURE 5:**
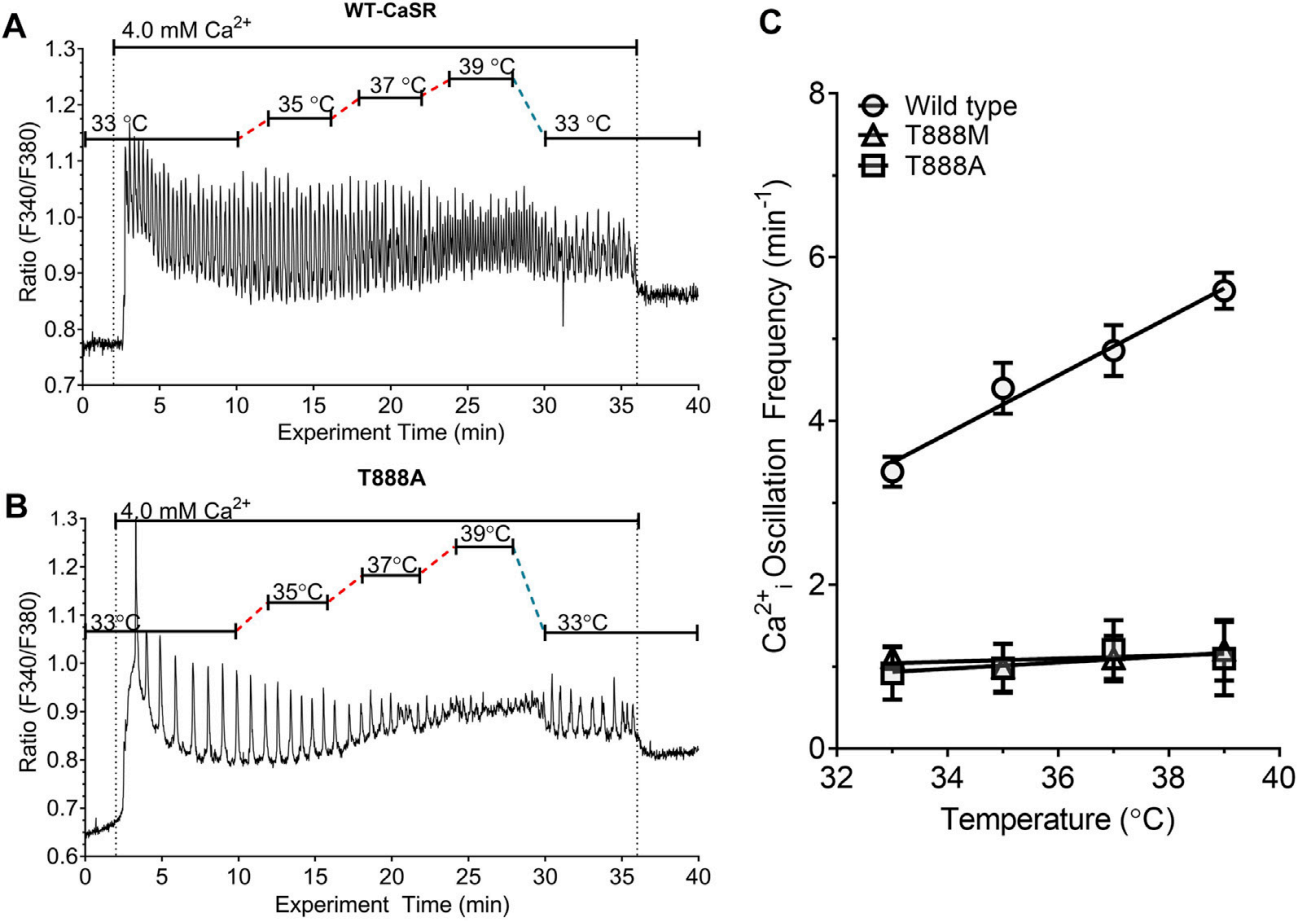
Effect of T888 mutants on temperature-dependent control of Ca^2+^_o_-induced Ca^2+^_i_ oscillations in transiently transfected HEK293 cells. HEK-293 cells were transiently transfected for 48 h with either the WT CaSR or CaSR mutants T888A or T888M then loaded with fura-2 AM and after equilibration to control Ca^2+^_o_ (0.5 mM) exposed to 4.0 mM Ca^2+^_o_ in a micro-fluorescence apparatus. Temperature was adjusted as described in the Methods section. Panels **(A, B)** show representative traces from single cells for WT and T888A. Panel **(C)** shows the relationships between temperature and Ca^2+^_i_ frequency for all three constructs. Temperature-dependent modulation of Ca^2+^_i_ oscillation frequency was not observed in cells transfected with either T888A or T888M. The results in C were obtained in four experiments in each case.

We next investigated whether temperature-dependent modulation of CaSR-mediated Ca^2+^_i_ oscillations requires CaSR residue T888. We transiently transfected HEK-293 cells with either wild-type CaSR or with one of the CaSR mutants T888A or T888M, in which the PKC-dependent phosphorylation site was eliminated. HEK-293 cells that were transiently transfected with wild-type CaSR were loaded with fura-2 then exposed to 4.0 mM Ca^2+^_o_ at various temperatures in the range 33°C—39°C. At 37°C the Ca^2+^_i_ oscillation frequency was 4.41 ± 0.74 min^−1^ (n = 4) emulating the responses observed in HEK-CaSR cells, in which the CaSR was expressed stably (Suppl. Figure 1). In addition, HEK-293 cells transiently transfected with the wild-type CaSR exhibited temperature-dependent modulation similar to that previously observed for HEK-CaSR cells (Figure 5A).

In HEK-293 cells that were transiently transfected with the activating mutations T888A (Figures 5B, C) or T888M (Figure 5C), the frequencies of Ca^2+^_o_-induced Ca^2+^_i_ oscillations were markedly suppressed. Thus, at 37°C in the presence of 4 mM Ca^2+^_o_ the Ca^2+^_i_ frequency in HEK-293 cells transiently transfected with T888M was 1.1 ± 0.28 min^−1^ (n = 7; *p* < 0.05), or with T888A was 1.03 ± 0.26 min^−1^ (n = 3; *p* < 0.05).

In cells that had been transiently transfected with T888M, an increase in Ca^2+^_o_ from 0.5 to 4.0 mM induced Ca^2+^_i_ oscillations in around 65% of cells (45/70 individual cells) and this response was independent of temperature over the range 31°C–41°C. Similar results were obtained with HEK-293 cells that were transiently transfected with T888A. Thus, temperature had a positive effect on the frequency of Ca^2+^_o_-induced Ca^2+^_i_ oscillations in HEK-293 cells transiently transfected with the wild-type CaSR but had no effect on Ca^2+^_i_ frequency in HEK-293 cells transiently transfected with either T888M or T888A (Figure 5C). In particular, slopes of the temperature-dependent responses observed in HEK-293 cells transiently transfected with WT, T888M and T888A and subsequently exposed to 4.0 mM Ca^2+^_o_ were, respectively: 0.36 ± 0.06 min^−1^°C^−1^; 0.01 ± 0.01 min^−1^°C^−1^; and 0.04 ± 0.05 min^−1^°C^−1^ (*p* < 0.01 for WT with respect to either T888M or T888A).

### PKC inhibition and temperature-dependent Ca^2+^_i_ oscillation frequency

**FIGURE 6:**
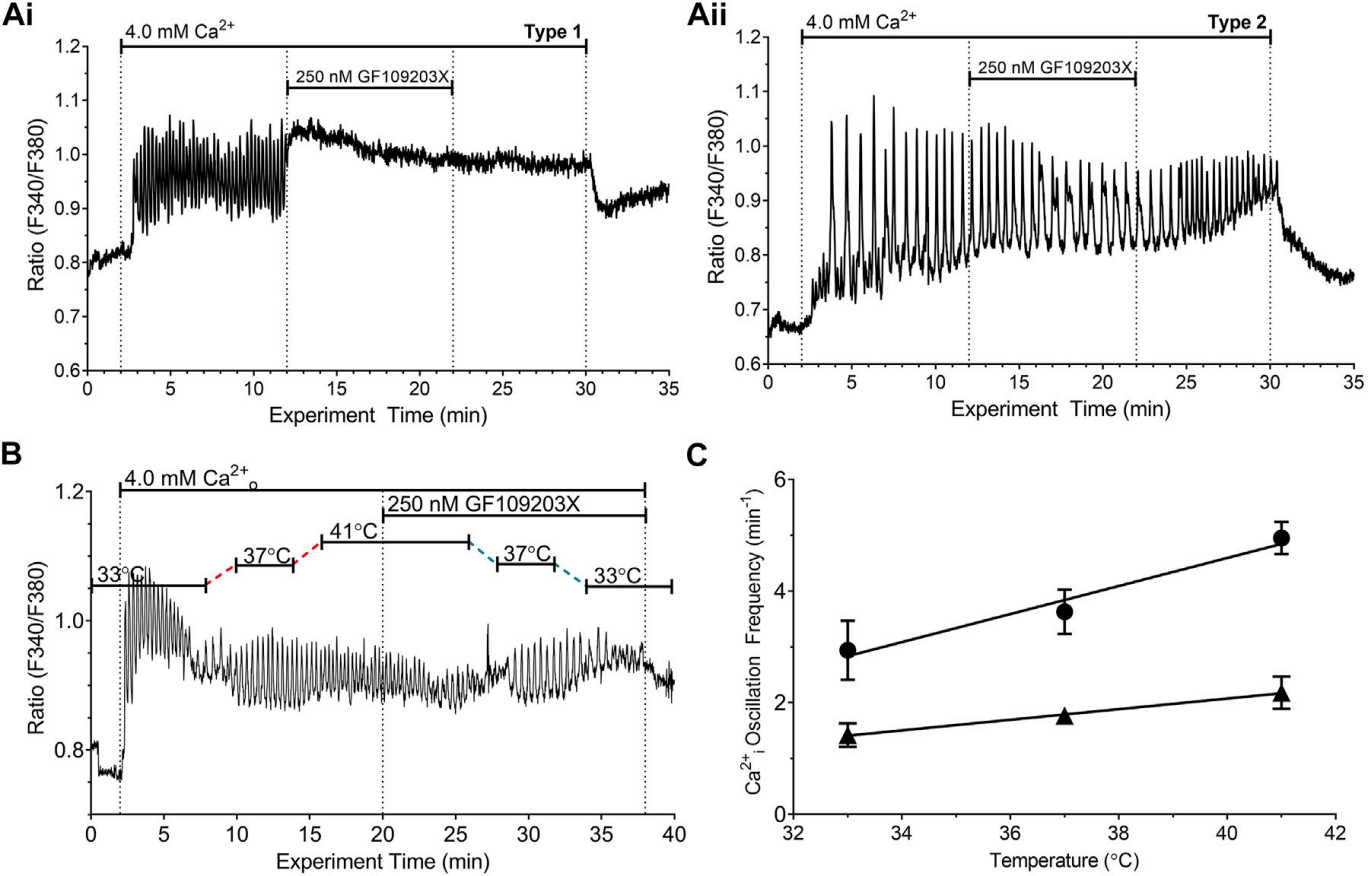
Effect of protein kinase C inhibitor on temperature-dependent modulation of the frequency of Ca^2+^_o_-induced Ca^2+^_i_ oscillations. HEK-CaSR cells were loaded with fura-2 AM then exposed to 4.0 mM Ca^2+^_o_ in the absence or presence of the PKC inhibitor GF109203X and the temperature was adjusted in the range 33°C—41°C. At the physiological control temperature, 37°C, GF109203X (250 nM) either (i) converted Ca^2+^_i_ oscillations into sustained responses (Type 1 response, ∼60% of cells; Panel **(Ai, ii)** reduced the frequency of Ca^2+^_i_ oscillations (Type 2 response, ∼40% of cells; Panel **(Aii)**. Panel **(B)** shows a representative trace from a single cell that was exposed to 4.0 mM Ca^2+^_o_ and three distinct temperatures (33, 37°C and 41°C). Ca^2+^_o_-induced Ca^2+^_i_ oscillations persisted upon exposure to GF109203X permitting analysis of the effect of the inhibitor as the temperature was re-adjusted to the control level. Panel **(C)** shows the relationships between temperature and Ca^2+^_i_ oscillation frequency for HEK-CaSR cells exposed to 4.0 mM Ca^2+^_o_ in the absence (circles) or presence (triangles) of GF109203X (250 nM). The data were obtained in six independent experiments.

Since phosphorylation of CaSR residue T888 is dependent on PKC (Bai et al., 1998), we next investigated the impact of GF109203X, a potent inhibitor of both α and β1 isoforms of PKC, which was previously reported to convert Ca^2+^_o_-induced Ca^2+^_i_ oscillations to sustained elevations in Ca^2+^_i_ in HEK-CaSR cells (Davies et al., 2007). Exposure of HEK-CaSR cells to 4.0 mM Ca^2+^_o_ at 37°C induced Ca^2+^_i_ oscillations in approximately 95% of cells as observed previously and GF109203X (250 nM) transformed the Ca^2+^_i_ responses from stable oscillations to sustained increases in ∼60% of cells, presumably as a consequence of the unopposed action of protein phosphatase activity (McCormick et al., 2010) (Type 1 response, Figure 6A2) 32 of 54 individual cells, n = 6). Ca^2+^_i_ oscillations continued in the remaining ∼40% of cells (Type 2 response, Figure 6A ii; 22 of 54 individual cells, n = 6) and the frequencies of Ca^2+^_i_ oscillations were lower than control at all temperatures tested (Figures 6B, C). Thus, in cells that continued to support Ca^2+^_i_ oscillations at 41°C in the presence of GF109203X, the Ca^2+^_i_ frequency was 2.18 ± 0.29 min^−1^ compared with 4.95 ± 0.29 min^−1^ (*p* < 0.0001) during the control period (Figures 6B, C). Similarly, in cells that continued to support Ca^2+^_i_ oscillations at 33°C, the Ca^2+^_i_ frequency was 1.42 ± 0.21 min^−1^ compared with 2.94 ± 0.53 min^−1^ (Figures 6B, C). Linear regression analysis demonstrated that GF109203X (250 nM) significantly reduced the slope of the relationship between temperature and Ca^2+^_i_ oscillation frequency from 0.25 ± 0.05 min^−1^°C^−1^ to 0.10 ± 0.01 min^−1^°C^−1^ (*p* < 0.001).

## Discussion

The CaSR is a key multi-modal sensor that mediates responses to multiple metabolic signals including activating divalent cations Ca^2+^ and Mg^2+^, L-amino acids, polyamines, small peptides, and pH (Conigrave et al., 2000a; Hofer and Brown, 2003; Feng et al., 2010). It is also inhibited by ionic strength (Quinn et al., 1998) and inorganic phosphate (Centeno et al., 2019). In the present study, we have demonstrated that CaSR-mediated signalling is sensitive to an additional physical modality, temperature *via* modulation of the frequency of Ca^2+^_o_-induced Ca^2+^_i_ oscillations. In HEK-CaSR cells we demonstrated that relatively small, stepwise increases in temperature over the pathophysiological range 31°C–41°C induced increases in the frequency of Ca^2+^_o_-induced Ca^2+^_i_ oscillations, but not in the amplitudes of the Ca^2+^_i_ responses or in their integrated values. Ca^2+^_i_ oscillation frequency was dependent on both Ca^2+^_o_ concentration and temperature, and the slope of the temperature-Ca^2+^_i_ frequency relationship increased as Ca^2+^_o_ increased from 2.0 to 4.0 mM. The Q_10_ values as temperature increased were around 2–3 (see below).

Unlike the wild-type CaSR, the CaSR/mGluR1/mGluR1 (C/G/G) chimeric receptor, which is composed of the human CaSR’s VFT domain fused to the rat mGluR1α cysteine-rich domain, heptahelical domain and intracellular C-terminus, exhibited high Ca^2+^_o_-induced low frequency Ca^2+^_i_ spikes (<1 min^−1^) that were insensitive to temperature (Figure 4). Although mGluR1 has a moderate degree of homology to the CaSR (∼30% identity in amino acid sequence) (Hu and Spiegel, 2007) mGluR1-mediated Ca^2+^_i_ responses do not take the form of PKC-dependent stable oscillations (Kawabata et al., 1998). The results demonstrate that the CaSR VFT domain is not sufficient for temperature-sensing, unlike its roles in nutrient-sensing (Mun et al., 2004) and implicate instead the receptor’s C-terminus.

The highly conserved PKC phosphorylation site T888 in the CaSR’s C-terminus is required for CaSR-mediated Ca^2+^_i_ oscillations (Bai et al., 1998; Young et al., 2002; Davies et al., 2007; McCormick et al., 2010). To investigate the role of T888 in temperature-sensing we used two non-phosphorylatable mutants, T888M and T888A. T888M was identified previously in a human kindred with autosomal dominant hypocalcemia (ADH) providing evidence that phosphorylation of T888 impairs 1) sustained elevations in Ca^2+^_i_ and 2) CaSR-mediated suppression of PTH secretion (Lazarus et al., 2011)*.* Both T888M and T888A were shown previously to exhibit left-ward shifts in the Ca^2+^_o_ concentration response curves for Ca^2+^_i_ mobilization relative to WT (Bai et al., 1998; Lazarus et al., 2011). A small subset of cells that expressed T888M or T888A exhibited low frequency Ca^2+^_i_ oscillations, apparently due to an inability to reach the threshold frequency required for the transition from Ca^2+^_i_ oscillations to sustained elevations in Ca^2+^_i_ (Bai et al., 1998; Lazarus et al., 2011). As predicted, cells transfected with T888M or T888A exhibited reduced frequencies of high Ca^2+^_o_ (4.0 mM)-induced Ca^2+^_i_ oscillations at 37°C and Ca^2+^_i_ frequency was not modulated by temperature (Figure 5).

Since PKC catalyses T888 phosphorylation we investigated the impact on temperature modulation of the potent, broad-spectrum PKC inhibitor GF109203X, whose spectrum of activity includes PKC-α, the isoform that mediates inhibitory control of Ca^2+^_i_ oscillations (Young et al., 2014). Consistent with a role for PKC in the control of Ca^2+^_i_ oscillations (Bai et al., 1998; Davies et al., 2007), GF109203X (250 nM) converted high Ca^2+^_o_-induced Ca^2+^_i_ oscillations to sustained elevations in Ca^2+^_i_ in around 60% of cells, presumably due to the unopposed action of protein phosphatase activity (McCormick et al., 2010). Approximately 40% of cells, however, retained Ca^2+^_i_ oscillations in the presence of GF109203X (Figure 6). In this subset of cells, the frequency of Ca^2+^_o_-induced Ca^2+^_i_ oscillations was significantly decreased and their sensitivity to temperature was also markedly reduced (Figure 6).

It is possible that mechanisms relating to the management of intracellular Ca^2+^ fluxes may contribute to temperature-related alterations in CaSR-dependent Ca^2+^_i_ oscillation frequency. Thus, store-operated Ca^2+^ channels including STIM1/Orai-1 (Parekh and Penner, 1997; Xiao et al., 2011), the endoplasmic reticulum Ca^2+^-ATPase (Shigekawa et al., 1976) and L-type voltage-gated Ca^2+^ channels (Peloquin et al., 2008) have all been reported to exhibit temperature sensitivity. In the present study, the loss of temperature dependence in cells transfected with the CaSR/mGluR1/mGluR1 chimeric receptor or T888 mutants, which disrupt the receptor’s primary PKC site, suggests that contributions from these effectors to CaSR-mediated temperature sensing are small.

The role of T888 phosphorylation and the impact of PKC inhibitor GF109203X points to a key role for PKC and possibly also protein phosphatase activity in CaSR-mediated temperature sensitivity. PKC and various protein phosphatases have been reported to exhibit temperature sensitivity (Sugiura et al., 2002; Kim et al., 2005; Chemin et al., 2007). Based on these considerations, any receptor that controls Ca^2+^_i_ oscillation frequency in a phosphorylation-dependent manner would be expected to adjust Ca^2+^_i_ frequency-dependent signaling outputs and attendant cell responses to the prevailing temperature. A phenomenon of this type operates in mesangial cells, in which activation of V1 vasopressin receptors (Jard et al., 1987) induces PKC-dependent Ca^2+^_i_ oscillations modulated by temperature over the range 24°C–37°C, with a Q_10_ value of around 3 (Hajjar and Bonventre, 1991), comparable to that observed for the CaSR in the present study. Interestingly, V1 vasopressin receptors were shown recently to modulate temperature sensing in the hypothalamus (Xu et al., 2018).

The present findings demonstrate that the CaSR contributes to temperature-dependent changes in cell function and are consistent with a role for the CaSR in mediating Ca^2+^_o_-dependent modulation of thermoregulation *in vivo.* Consistent with this idea, the CaSR is expressed at high levels in the anterior preoptic nucleus of the hypothalamus (Yano et al., 2004), which participates in central thermoregulation and is sensitive to acute changes in core and local temperatures (Schmid and Pierau, 1993; Boulant, 2000; Tabarean, 2018). In addition, Ca^2+^_o_ (0.9–1.5 mM) negatively modulates core temperature in various species (Hanegan and Williams, 1973; Nielsen, 1974; Myers et al., 1976), and also negatively modulates the firing rates and temperature coefficients of anterior preoptic neurons (Schmid and Pierau, 1993). Finally, in the context of type-1 Autosomal Dominant Hypocalcemia (ADH-1), arising from activating mutations of the CaSR ((Pollak et al., 1994); review: (Egbuna and Brown, 2008)) and in which there is enhanced Ca^2+^_o_-induced inhibition of Ca^2+^ reabsorption and attendant hypercalciuria, there is an increased risk of febrile convulsions in childhood, raising the possibility that acute drops in CNS extracellular fluid Ca^2+^_o_ due to acute exacerbations of hypercalciuria might disable CaSR-mediated negative control of core body temperature, either inducing a fever or exacerbating a co-incident fever resulting in disordered CNS neuronal activity and attendant convulsions. Renal hypercalciuria arising from other genetic disorders has also been linked to febrile convulsions in childhood (Papadimitriou et al., 2001; Gorabi et al., 2018), presumably secondary to acute drops in plasma Ca^2+^ concentration.

In considering appropriate models for investigating the pathophysiological significance of our findings, in preliminary experiments we screened a human parathyroid cell preparation, which expresses the CaSR endogenously, for secretory responses to temperature. We observed that switching from physiological temperature (37°C) to a low temperature (30°C) reversibly lowered PTH secretion, as expected for enzyme-dependent exocytosis from membrane-bound vesicles, and also exhibited an apparent, low temperature-dependent impairment of the CaSR-mediated inhibitory response, as Ca^2+^_o_ was raised from 0.8 mM to 1.2 mM. Conversely, when the temperature was raised from 35°C to 38°C, we observed enhanced PTH secretion at a Ca^2+^_o_ concentration of 0.8 mM, together with enhanced suppression of PTH secretion at a Ca^2+^_o_ concentration of 1.1 mM. However, the cell preparation failed to recover to its baseline PTH secretory rate upon return to the control temperature (Supplementary Figure S2). Although the results are consistent with the temperature-sensing properties we observed in the present study, the failure of the parathyroid cell preparation to recover normal secretory control following large excursions in temperature leads us to conclude that animal rather than cell models are likely to be more informative. One such model would be a tissue-selective CaSR-null mouse in which upon deletion of the CaSR from the anterior preoptic nucleus, loss of Ca^2+^_o_-dependent negative modulation of core temperature would be expected.

In the absence of readily available animal models, it is feasible, nevertheless, to identify the CaSR as a novel, functionally relevant, thermoregulatory sensor based on *three key conditions* (Wang and Siemens, 2015): 1) the candidate protein should exhibit thermoregulatory properties; 2) the candidate protein should be expressed in temperature-sensing CNS organs such as the anterior preoptic nucleus; and 3) thermoregulatory mechanisms should be impaired in one or more disorders related to the candidate gene/protein. Based on the outcomes of the present study and on results reported in the literature, the CaSR satisfies all three criteria. Thus, it exhibits thermoregulatory properties (Criterion-i), it is expressed in a key temperature-sensing organ, the anterior preoptic nucleus (Yano et al., 2004) (Criterion-ii), and it has been linked to a genetic disorder of thermoregulation (infantile febrile convulsions) in human cases of ADH (review: (Egbuna and Brown, 2008)) (Criterion-iii).

The Q_10_ temperature coefficient values for the CaSR in the current experimental system lay in the range 2.0—3.0, consistent with the behavior of enzymes (Kotov et al., 2007), and point to control by temperature-sensitive protein kinases and/or phosphatases, as noted above. This raises the possibility that the CaSR modulates the behavior of other temperature sensors. Temperature sensing has been ascribed previously to several transient receptor potential (TRP) ion channels. These ThermoTRPs include four heat-activated channels (TRPV 1—4) and three cold-activated channels (TRPM2, TRPM8 and TRPA1) (Islas and Emir, 2017). Several mammalian TRP channels have Q_10_ temperature coefficient values (defined by the fold-change of effector output as temperature *increases* by 10°C) > 5 (warm sensitive) or <0.2 (cold sensitive) (Wang and Siemens, 2015). Furthermore, some high temperature-sensitive TRP channels, such as TRPV3 and TRPV4, exhibit Q_10_ values around 20 (Baylie and Brayden, 2011). Whether the CaSR modulates the activation states of one or more ThermoTRP channels is unknown, however, TRPV4, which is sensitive to the temperature range 25°C–34°C (Almeida et al., 2015), is co-expressed along with the CaSR in the anterior preoptic hypothalamus and the CaSR interacts with and activates TRPV4/TRPC1 heteromeric channels (Greenberg et al., 2017), as well as TRPC1 in the parathyroid as reported recently (Onopiuk et al., 2020).

The identification of the CaSR’s temperature-sensing properties extends its repertoire of physical and chemical sensing properties (reviews: (Conigrave et al., 2000a; Conigrave and Hampson, 2006; Conigrave and Hampson, 2010; Feng et al., 2010). The results underscore the role of the receptor in systemic homeostasis and in the metabolism of specific local compartments.

In conclusion, we have demonstrated that the CaSR responds to changes in temperature in the pathophysiological range 31°C–41°C. Temperature positively and reversibly modulated Ca^2+^_i_ oscillation frequency, and these responses were supported by PKC-dependent phosphorylation and, presumably, dephosphorylation of the CaSR C-terminal residue T888. Expression of the CaSR in thermoregulatory organs of the hypothalamus, observations that Ca^2+^_o_ negatively modulates core body temperature together with the firing rates of thermoregulatory neurons, the existence of interactions between Ca^2+^_o_ and the CaSR on the behavior of thermoregulatory ion channels, and the recognised impact of genetic disorders of calcium metabolism and/or the CaSR on thermoregulation and CNS function are all consistent with the hypothesis that the CaSR modulates thermal regulation *in vivo*.

## Data Availability

The datasets presented in this study can be found in online repositories. The names of the repository/repositories and accession number(s) can be found below: https://researchdata.edu.au/effect-temperature-calcium-oscillations-dataset/267924.
