# Supplementary material for: Temperature sensing by the calcium-sensing receptor
Source: Front Physiol. 2023 Feb 2;14:1117352. doi: 10.3389/fphys.2023.1117352 (PMC9931745; doi:10.3389/fphys.2023.1117352)
Supplement: Supplementary file 1 [file Table1.docx]

# **Supplemental**

**Suppl. Table 1: Comparisons between set temperatures and temperatures of effluent solutions using the heated microscope stage**

Perifusion solutions were pre-equilibrated using a circulating water bath and temperature losses were minimized using a heated microscope stage. The data were obtained in three experiments.

| **Set Temperature (°C)** | **Effluent Temperature (°C)** |
| --- | --- |
| 31 | 31.1 ± 0.2 |
| 33 | 33.4 ± 0.4 |
| 35 | 34.9 ± 0.1 |
| 37 | 37.1 ± 0.1 |
| 39 | 38.8 ± 0.3 |
| 41 | 40.7 ± 0.4 |
