# Supplementary material for: Temperature sensing by the calcium-sensing receptor
Source: Front Physiol. 2023 Feb 2;14:1117352. doi: 10.3389/fphys.2023.1117352 (PMC9931745; doi:10.3389/fphys.2023.1117352)
Supplement: Supplementary file 2 [file Image2.pdf]

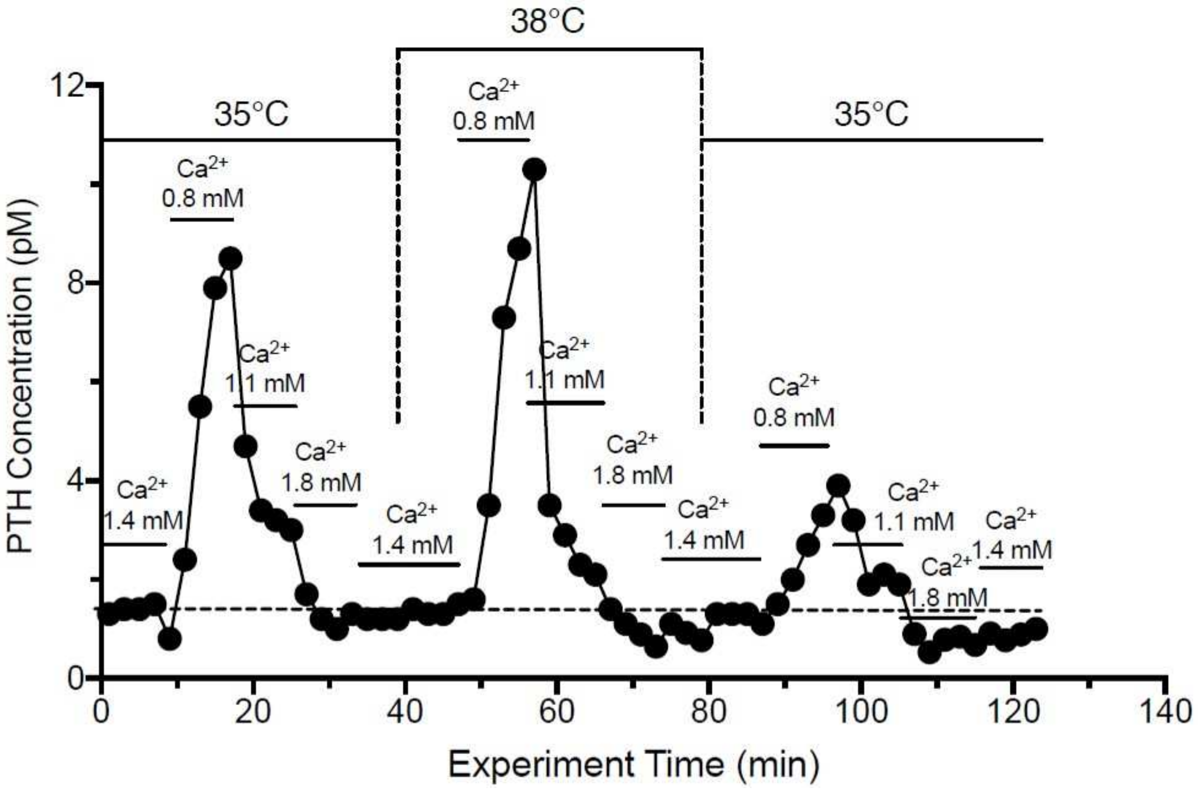

Suppl. Fig 2. Effect of temperature on Ca^{2+}-sensitive PTH secretion

A preliminary experiment in which we investigated temperature-dependent modulation of Ca^{2+}_o-dependent PTH secretion from normal human parathyroid cells. After an initial period in which the cells were exposed to a sub-physiological temperature, 35°C, for approximately 40 min, the temperature was raised to a supraphysiological temperature, 38°C for a further 40 min, before being adjusted back to 35°C, for a final period of approximately 45 min. Within each of the three periods in which the temperature was fixed, Ca^{2+}_o was adjusted initially to 1.4 mM, at the upper limit of the physiological concentration range (1.1–1.4 mM). It was then lowered to a sub-physiological concentration, 0.8 mM and then adjusted in sequence to 1.1 mM, 1.8 mM, and finally 1.4 mM once more. As expected, we observed Ca^{2+}_o-dependent suppression of PTH secretion in all three major periods during which the temperature was fixed. Interestingly, following the

transition to the high temperature, 38°C, we observed both an exaggerated increase in PTH secretion at low Ca^{2+}_o and an exaggerated decrease in PTH secretion at the Ca^{2+}_o level corresponding to the lower limit of the normal range, 1.1 mM, as well as at higher Ca^{2+}_o levels. The enhanced suppression of PTH secretion at raised Ca^{2+}_o in the presence of high temperature is consistent with our observations that the CaSR exhibits temperature-sensing properties and that increased temperature promotes the receptor's sensitivity to Ca^{2+}_o. Following exposure to the high temperature, 38°C , and upon the return to baseline, 35°C, however, the cells were unable to recover baseline PTH secretion levels, which remained continuously suppressed despite exhibiting residual Ca^{2+}_o sensitivity.
