# Supplementary material for: Temperature sensing by the calcium-sensing receptor
Source: Front Physiol. 2023 Feb 2;14:1117352. doi: 10.3389/fphys.2023.1117352 (PMC9931745; doi:10.3389/fphys.2023.1117352)
Supplement: Supplementary file 3 [file Image1.pdf]

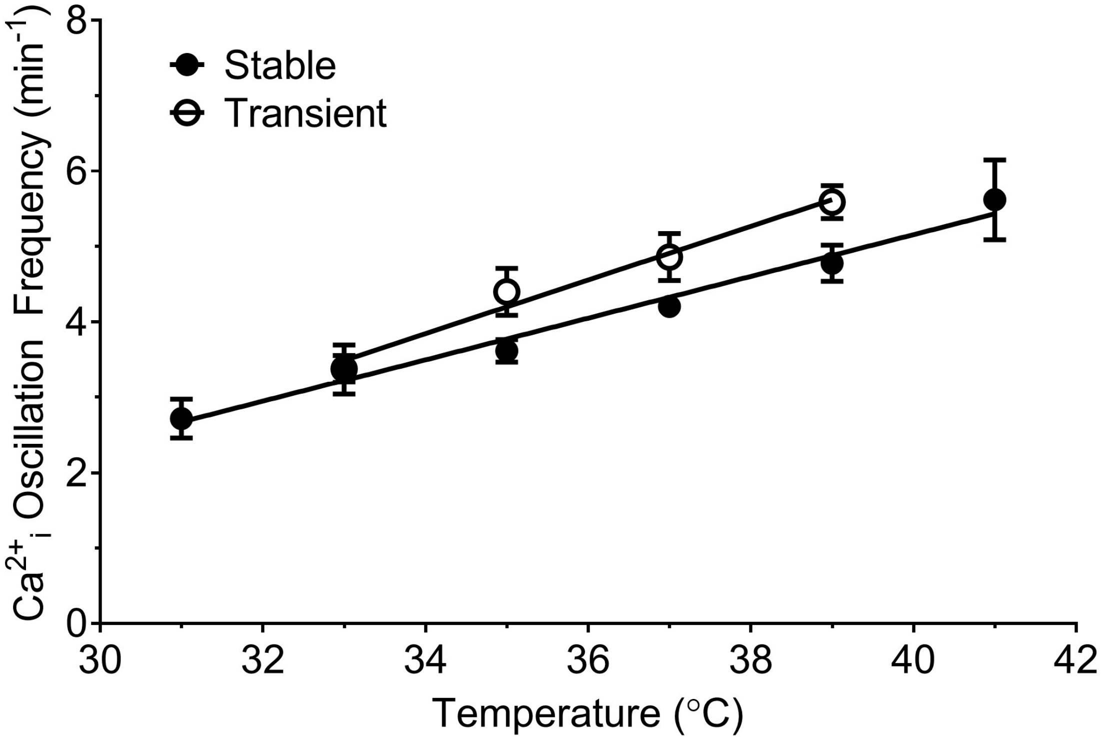

Suppl. Fig 1. Relationships between temperature and $Ca^{2+}{}_o$-induced $Ca^{2+}{}_i$ frequency in HEK-293 cells transfected transiently or stably with the wild-type CaSR

Temperature-dependent modulation of $Ca^{2+}{}_o$-induced $Ca^{2+}{}_i$ oscillation frequency was compared in HEK-293 cells that had been transiently or stably transfected with the wild-type CaSR. Stable transfectants are referred to as 'HEK-CaSR cells'. After a period of equilibration at baseline $Ca^{2+}{}_o$ (0.5 mM), the cells were exposed to a sub-maximal $Ca^{2+}{}_o$ (4.0 mM) and then exposed to various temperatures. Similar temperature-$Ca^{2+}{}_i$ frequency relationships were observed in both cases.
